# Production of dental prosthetics in the SUS in Brazilian older population and impact of the covid-19 pandemic

**DOI:** 10.11606/s1518-8787.2023057004828

**Published:** 2023-08-03

**Authors:** Manuella Fluck Vieira, Patrícia Schunck Alferes Marques, Daniela de Rossi Figueiredo, Daniela Lemos Carcereri, Andreia Morales Cascaes

**Affiliations:** I Universidade Federal de Santa Catarina Programa de Pós-graduação em Odontologia Florianópolis SC Brasil Universidade Federal de Santa Catarina. Programa de Pós-graduação em Odontologia. Florianópolis, SC, Brasil; II Faculdade São Leopoldo Mandic Programa de Pós-graduação em Odontologia Campinas SP Brasil Faculdade São Leopoldo Mandic. Programa de Pós-graduação em Odontologia. Campinas, SP, Brasil; III Universidade do Sul de Santa Catarina Departamento de Odontologia Florianópolis SC Brasil Universidade do Sul de Santa Catarina. Departamento de Odontologia. Florianópolis, SC, Brasil; IV Universidade Federal de Santa Catarina Departamento de Odontologia Florianópolis SC Brasil Universidade Federal de Santa Catarina. Departamento de Odontologia. Florianópolis, SC, Brasil; V Universidade Federal de Santa Catarina Departamento de Saúde Pública Florianópolis SC Brasil Universidade Federal de Santa Catarina. Departamento de Saúde Pública. Florianópolis, SC, Brasil

**Keywords:** Dental Prosthetics, Unified Health System, Elderly, Brazil, COVID-19

## Abstract

**OBJECTIVE:**

Describe the trends in the production of dental prosthetics by the Unified Health System (SUS) in older people aged 60 years or older in Brazil and country regions from 2010 to 2019 and the impact of the covid-19 pandemic on the expected production for 2020 and 2021.

**METHODS:**

A time series study using secondary data from the SUS database (Datasus-Tabnet) and the *Instituto Brasileiro de Geografia e Estatística* (Brazilian Institute of Geography and Statistics - IBGE) from 2010 to 2021. Age-standardized rates were calculated for Brazil and regions for each year analyzed. Generalized linear regressions estimated production trends using the Prais-Winstein estimation method.

**RESULTS:**

A growth trend occurred in the standardized production rate of complete dentures and other prosthetics per 100,000 inhabitants in Brazil and all country regions. The increase in the production of complete dentures was higher in the Northeast region (50.3%/year) and lower in the North region (19.1%/year). Trends in the production of other prosthetics were higher in the Southeast region (120.7%/year) and lower in the North region (24.5%/year). The output of prosthetics for both groups decreased in the pandemic years. In 2020, the relative difference ranged from -36.4% (North) to -61.7% (Northeast) for producing complete dentures and from -17.9% (North) to -68.4% (Northeast) for other prosthetics. In 2021, standardized rates and total production increased compared to the previous year. However, compared with expected values, the differences were close to those in 2020.

**CONCLUSION:**

Policies aimed at producing complete dentures and other prosthetics have been increasing. However, production remains far from the population’s needs, and there is no equity in providing services. The covid-19 pandemic negatively impacted the production of dental prosthetics by SUS.

## INTRODUCTION

The total loss of teeth (edentulism) is still accepted by many as a natural phenomenon of aging^1^. However, it represents the outcome of the history of dental treatments (or their absence) and the dental diseases of the individual’s life^2^. It has a complex etiology, interrelation with biological, social, and behavioral factors, with caries and periodontal disease as its main precursors^3^. In 2010, 158 million people were edentulous, constituting 2.3% of the world’s population. With age, there is a gradual increase in the prevalence of tooth loss, with a peak incidence at 65 years of age^4^. Population aging at an accelerated pace changes age composition, impacting health systems.

Older people accounted for 10.7% of the Brazilian population in 2010, rising to 14.3% in 2020^5^. According to the National Oral Health Surveys (SB-Brasil)^6,7^, the percentage of older adults between 65 and 74 years old with missing teeth remained in the range of 90% between 2003 and 2010. According to the 2010 survey, in this age group, 38.3% needed a complete denture^7^. Furthermore, in a worldwide comparison, Brazil had the highest age-standardized prevalence of severe tooth loss in 2010, corresponding to 3.9%^4^. Concerning the quality of life, tooth loss compromises the daily routine, brings functional and social consequences^8,9^, affects self-esteem^8^, increases the risk of nutritional deficit^9^, can reflect on individual perceptions, including self-perception of the need for dental treatment^10^, and beg a risk factor for accelerated cognitive aging^11^. Edentulous individuals without dental prosthetics have a more significant negative impact on their quality of life than users of dental prosthetics^9^.

In Brazil, prosthetic rehabilitation services occur in the Unified Health System (SUS) through the Basic Health Units (UBS)^12^, which are responsible for primary health care, Dental Specialty Centers (CEOs), and Regional Dental Prosthetics Laboratories (LRPDs). It is up to health managers to analyze the need and feasibility and define the implementation, types, and quantity of dental prosthetics to be produced^13^. An uneven distribution in the supply of these services occurs in the country, which is confirmed by the monthly rates of delivery of prosthetics, rates of use, and supply of CEOs and LRPDs in the regions^14,15^. In March 2020, due to the covid-19 pandemic, several services in the SUS, including elective dental care, were suspended, with only emergency dental care remaining^16^. Despite the availability of data, there is a lack of studies on the prosthetic production of the SUS over long periods and the impact caused by the covid-19 pandemic in the area. This study aimed to describe the trends in the production of dental prosthetics by the SUS in older people aged 60 years or older in Brazil and regions from 2010 to 2019, in addition to estimating the impact caused by the covid-19 pandemic on the expected production for 2020 and 2021.

## METHODS

This time series study used secondary data from the SUS database (Datasus-Tabnet)^17^ and the *Instituto Brasileiro de Geografia e Estatística* (IBGE)^5^ from 2010 to 2021.

We obtained the production of dental prosthetics from the records of the SUS Outpatient Information System (SIA/SUS) on the Datasus-Tabnet website^17^, classified according to the place of residence, age group, and year of data processing in the system. In obtaining the data, we opted for the place of residence rather than the care location, as only the former enabled us to choose the users’ age range. The registered production of dental prosthetics considers the quantity approved for payment by the secretariats, as recorded in the Management System for the Table of Procedures, Medications, Orthotics, Prosthetics, and Special Materials of the SUS (Sigtap)^18^. Two large groups encompassed the selected procedures, namely: complete dentures group (07.01.07.012-9: total mandibular prosthesis; 07.01.07.013-7: complete maxillary prosthesis) and other dentures group (07.01.07.009-9: removable partial mandibular prosthesis; 07.01.07.010-2: removable partial maxillary prosthesis; 07.01.07.014-5: fixed/adhesive coronary/intraradicular prostheses per element; 07.01.07.015: dental prosthesis on implant).

We obtained data on the number of older people residing in the Brazilian states from the Population Projections for Brazil and Federation Units by Sex and Age: 2000–2030, from the IBGE^5^, whose data are available on the Datasus-Tabnet website^17^. This study considered individuals aged 60 years or older to be older people^19^.

For data analysis, we calculated crude and age-standardized rates for Brazil and each region in the analyzed years based on the population and production found in the period. The calculation of the gross rate considered the production of prosthetics divided by the actual older population in each location multiplied by 100,000 inhabitants. The rate standardized by age used the direct method^20^, with age ranges every five years, taking as standard the average age structure of the older population in the country in the analyzed period multiplied by 100,000 inhabitants.

We organized and analyzed data in spreadsheets in Microsoft Excel. We used the program’s tools and formulas to generate tables and figures. We used the Stata 14.2 program (StataCorp LP, College Station, United States) with generalized linear regressions using the Prais-Winstein estimation method^21^ for time series analysis of prosthesis production. Estimates of prosthesis production trends considered the years 2010 to 2019, considering that 2020 and 2021 showed a significant drop in the production of prosthetics due to the covid-19 pandemic. We used the following formulas to obtain annual percentage variation (VPA) = 100*(-1 + 10^b) and 95%CI = 100*(-1 + 10^[b ± t*EP])^21^. The trend was considered increasing when the coefficients were positive, decreasing when negative, and stable when the regression coefficients were not significantly different from zero (p > 0.05). The VPA calculation from 2010 to 2019 estimated the expected production for 2020 and 2021 as if there had been no pandemic. The absolute difference between the observed and expected production rate for 2020 is the observed production rate in 2020 minus the expected production rate in 2020. The relative difference between the observed and expected production rate for 2020 was calculated by the formula = [(observed production rate in 2020 - expected production rate in 2020) -1] × 100. We used a similar calculation to obtain the estimates for 2021.

## RESULTS

Table 1 shows the evolution of the production of complete dentures for the older people aged 60 years or older, from 2010 to 2021, in Brazil and regions. A growth trend occurred in Brazil’s standardized production rate of complete dentures per 100,000 inhabitants and in all regions of the country. This increase was higher in the Northeast region (VPA = 50.3%) and lower in the North region (VPA = 19.1%). The production of complete dentures decreased in 2020 and rose again in 2021. The absolute difference between the observed and expected standardized production rates for 2020 ranged from -333.0 (North) to -1,314.1 (Northeast). The relative difference in decline ranged from -36.4% (North) to -61.7% (Northeast). Despite the increase in the standardized rate found in 2021, the absolute and relative differences between the expected and the observed in that year were close and, for the most part, more significant than those found in 2020.

**Table 1:** Evolution of the production of complete dentures in older people aged 60 years or older, by region of Brazil and overall. Datasus, Brazil, 2010–2021.

Variables	North	Northeast	Southeast	South	Midwest	Brazil
Standardized production rate per year						
2010	331.0	251.7	268.3	424.3	408.1	301.5
2011	380.2	476.6	469.5	539.0	565.4	483.9
2012	460.7	618.0	619.3	624.1	782.1	621.2
2013	546.1	832.5	683.0	623.0	745.9	706.0
2014	582.6	1,188.8	751.5	763.1	918.5	860.9
2015	561.6	1,071.8	777.8	851.9	897.0	856.7
2016	430.0	1,096.7	775.9	878.2	929.4	860.7
2017	439.2	1,049.4	678.3	854.0	809.2	791.1
2018	576.7	1,292.6	667.1	881.7	979.3	865.6
2019	767.3	1,416.8	747.3	943.1	1,078.7	959.2
2020	580.9	815.3	392.5	558.5	715.9	550.2
2021	606.3	985.2	528.7	657.0	840.3	680.8
Prais-Winsten regression (2010 to 2019)						
β1 coefficient (95%CI)	0.076 (0.009–0.142)	0.177 (0.077–0.277)	0.101 (0.005–0.198)	0.084 (0.050–0.118)	0.089 (0.037–0.141)	0.119 (0.039–0.199)
VPA (95%CI)	19.1 (2.2–38.8)	50.3 (19.5–89.1)	26.3 (1.1–57.7)	21.4 (12.2–31.3)	22.7 (8.8–38.5)	31.5 (9.5–58.0)
p-value	0.030	0.003	0.042	0.000	0.004	0.009
Expected production for 2020						
Expected production rate	913.9	2,129.5	943.8	1,144.8	1,323.9	1,261.7
Absolute difference (rate)	-333.0	-1,314.1	-551.3	-586.2	-608.0	-711.5
Relative difference (%)	-36.4	-61.7	-58.4	-51.2	-45.9	-56.4
Expected production for 2021						
Expected production rate	1,088.5	3,200.6	1,192.0	1,389.6	1,625.0	1,659.7
Absolute difference (rate)	-482.2	-2,215.4	-663.3	-732.6	-784.7	-978.9
Relative difference (%)	-44.3	-69.7	-55.6	-52.7	-48.3	-59.0

95%CI: 95% confidence interval; VPA: annual percentage variation.

Note: production rate standardized by the direct method. Expected production rate for 2020 = [(2019 production rate × VPA)/100] + 2019 production rate. Absolute difference = observed production rate in 2020 - expected production rate in 2020. Relative difference = [(observed production rate in 2020 - expected production rate in 2020) - 1] × 100. We used a similar calculation to obtain the estimates for 2021.

Table 2 shows the evolution of the production of other prosthetics in older people aged 60 years or older from 2010 to 2021 in Brazil and regions. Except for the North region, standardized production rates increased over the years, showing stability. The Southeast (VPA = 120.7%) and Northeast (VPA = 92.1%) regions had the most significant trends of increase in the production of other prosthetics. The absolute difference between the observed and expected standardized rates for 2020 was greatest in the Northeast (-550.5) and Midwest (-620.6) regions. The relative difference ranged from -17.9% (North) to -68.4% (Northeast). For 2021, the differences increased; the highest absolute values found were Northeast (-1,208.2) and Midwest (-1,656.8). The relative difference ranged from -33.2% (North) to -82.4% (Midwest).

**Table 2:** Evolution of the production of other prosthetics in older people aged 60 or over, by region of Brazil and overall. Datasus, Brazil, 2010–2021.

Variables	North	Northeast	Southeast	South	Midwest	Brazil
Standardized production rate per year						
2010	67.7	31.3	53.6	49.7	13.9	45.5
2011	97.5	59.4	81.6	105.5	53.2	79
2012	120.3	82.8	113.8	182	142.9	119.5
2013	158.2	145.5	137.1	213	187.1	155.8
2014	108.4	240.9	167.4	269	213.1	201.5
2015	94.8	245.1	197.9	258.5	263.8	217.8
2016	68.5	278.5	218	309.5	294	244.2
2017	95	300.8	210.6	326.7	259.4	247.9
2018	139	368.1	227.8	354.3	362.8	285.5
2019	215	418.8	263.2	398.8	412.7	328.9
2020	219.9	254	147.6	253.7	290.2	203.5
2021	222.7	337.2	216.6	297.1	353	267.5
Prais-Winsten regression (2010 to 2019)						
β1 coefficient (95%CI)	0.095 (-0.032 to 0.223)	0.284 (0.166 to 0.401)	0.174 (0.099 to 0.248)	0.214 (0.100 to 0.329)	0.344 (0.130 to 0.557)	0.215 (0.119 to 0.311)
VPA (95%CI)	24.5 (-7.2 to 67.1)	92.1 (46.5 to 151.9)	49.1 (25.5 to 77.1)	63.8 (25.9 to 113.2)	120.7 (35.0 to 260.7)	64.1 (31.7 to 104.5)
p-value	0.124	0.001	0.001	0.003	0.006	0.001
Expected production for 2020						
Expected production rate	267.7	804.5	392.5	653.3	910.8	539.7
Absolute difference (rate)	-47.8	-550.5	-244.9	-399.6	-620.6	-336.2
Relative difference (%)	-17.9	-68.4	-62.4	-61.2	-68.1	-62.3
Expected production for 2021						
Expected production rate	333.3	1,545.4	585.3	1,070.2	2,009.8	885.6
Absolute difference (rate)	-110.6	-1,208.2	-368.7	-773.1	-1,656.8	-618.1
Relative difference (%)	-33.2	-78.2	-63	-72.2	-82.4	-69.8

95%CI: 95% confidence interval; VPA: annual percentage variation.

Note: production rate standardized by the direct method. Expected production rate for 2020 = [(2019 production rate × VPA)/100] + 2019 production rate. Absolute difference = observed production rate in 2020 - expected production rate in 2020. Relative difference = [(observed production rate in 2020 - expected production rate in 2020) - 1] × 100. We used a similar calculation to obtain the estimates for 2021.

**Figure 1:**
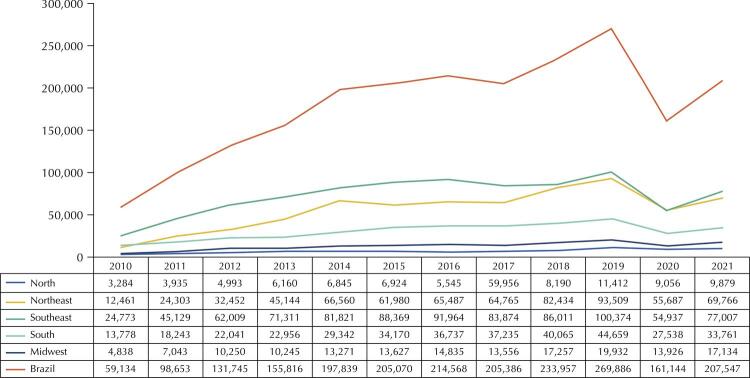
Total production of complete dentures in older people aged 60 years or older, by region of Brazil and overall. Datasus, Brazil, 2010–2021.

**Figure 2:**
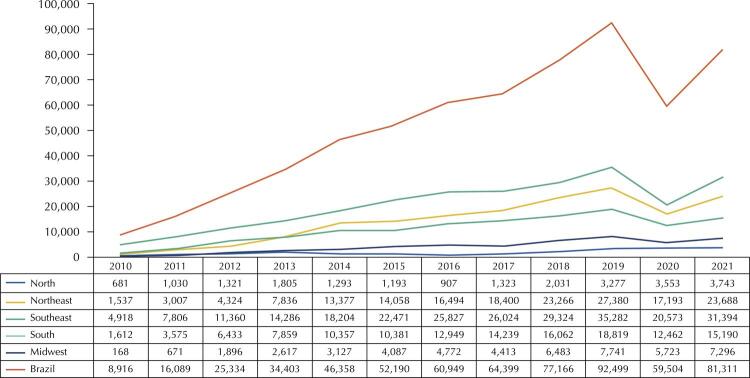
Total production of other prostheses in older people aged 60 years or older, by region of Brazil and overall. Datasus, Brazil, 2010–2021.

Figures 1 and 2 show the total production of complete dentures and other prosthetics from 2010 to 2021 in Brazil and its regions. The national output of complete dentures showed almost constant growth, starting with 59,134 prosthetics in 2010, reaching 269,886 in 2019, falling to 161,144 in 2020, and rising again in 2021, with 207,547 produced (Figure 1). The national production of other prosthetics increased until 2019, starting with 8,916 in 2010, reaching 92,499 in 2019, falling to 59,504 in 2020, and increasing again in 2021, with 81,311 produced (Figure 2). In most years, the total production of both analyzed groups was lower in the North region and higher in the Southeast.

## DISCUSSION

This study sought to analyze trends in the production of dental prosthetics by the SUS in older people aged 60 years or older in Brazil and regions and to observe the impact caused by the covid-19 pandemic. The main results point to the growing production of prosthetics in the country until 2019, the year before the pandemic. The complete dentures group had a higher output when compared to the other dentures group. In Brazil and all regions, in both analyzed groups, the impact of the pandemic on the reduction of prosthetic production was significant.

This study used and valued public and available data, producing relevant knowledge for the management of public oral health nationwide. As for the study’s limitations, the database used stands out since the SIA-SUS has no standardized records, and there is still a lack of studies assessing data reliability about dental procedures. The system is often used for billing procedures performed, leading to the possibility of over-registration.

Concerning the annual increase in the total production of prosthetics, the results are related to public policies aimed at expanding the supply of these procedures. Inserted in 2000 in the *Programa Saúde da Família* (PSF - Family Health Program), dentistry provided low-resolution assistance, depending on merely curative and mutilating services^22^. In 2004, with the *Programa Brasil Sorridente* (Smiling Brazil Program) implementation, oral health services were organized and planned^22^. Until the program’s launch, only 2.8% of dental procedures performed in the SUS were specialized treatments^23^. Since then, installation and maintenance of prosthetics can be carried out in primary health care^12^ and in CEOs, and the production of prosthetics in LRPDs and CEOs, except prosthetics on implants, which occur only in the latter^24^. In 2009, the ordinance of criteria, standards, and requirements for the implementation and qualification of CEOs and LRPDs had been created for five years^13^. It is important to note that, overall, in addition to annual growth in total production, standardized rates also increased over time.

The findings of this study point to inequalities in the production of prosthetics between Brazilian regions. Several factors may be related, such as how LRPDs and CEOs are implemented and managed^13,25^, the number of accredited units^26^, the minimum number of procedures performed^13^, socioeconomic and demographic differences^2^, and need differences in the regions^7^. The North region, followed by the Midwest, has the lowest number of CEOs and LRPDs^15,26^. The Northeast has the bigger numbers, followed by the Southeast^15,26^. These data partially support the total production found in this study, especially concerning the production of complete dentures.

The insertion date of the types of procedures and their outpatient values may interfere with the differences between the analyzed prosthesis groups. For example, prosthetics on implants started to be offered only at the end of 2010^18^. Furthermore, this type of treatment can only be performed in CEOs, leaving the municipal manager to request the Ministry of Health (MS) to offer these services^27^. In addition to the greater complexity involved in the production of the other prosthetics, issues related to the cost of the procedures may explain the lower output of this group. Since its implementation in the system, the implant prosthesis has a value of 300.00 BRL, and the other procedures, since 2012, have a value of 150.00 BRL^18^. However, the laboratory cost is known to be different between prosthetics, and complete dentures generally have a lower cost, ease of installation, production, and maintenance, in addition to a greater degree of severity of the oral condition. Such factors may be related to the higher production found in that group.

Regarding the minimum production of procedures performed, most LRPDs in the country are accredited with producing between 20 and 50 prosthetics per month^26^. The specialty of dental prosthetics is not mandatory in CEOs^13^. These factors support the low standardized production rates found in this study.

According to data from SB Brasil 2010^7^, prosthesis needs vary between regions and analyzed groups. For the need for complete dentures, in decreasing order, the last national survey presented the following percentages: 46.5% (Midwest), 45.6% (North), 41.1% (Northeast), 39% (Southeast), and 27.3% (South). These needs, when related to the results of this study, demonstrate production inequality, mainly concerning the North region. Regarding the other prosthetics, the percentages of need were: 66.1% (South), 61.7% (Northeast), 58% (Southeast), 56.4% (Midwest), and 56.3% (North)^7^. Despite having more significant needs, the group of other prosthetics has lower production. Still, the amount of LRPD per region does not follow the order of need for dental prosthesis^14^. Notably, regional differences in both groups tend to increase, leading to greater contrast due to increased production not following the order of necessity.

In national parameters, 38.3% of the older people aged 65–74 needed a complete prosthesis, and 59.3% required one of the types in the other prosthetics^7^. Such values represented in absolute numbers, respectively, 7,507,510 and 11,623,899 older Brazilians aged 60 years or older in 2010, according to the Population Projections for Brazil and Federation Units by Sex and Age: 2000–2030. In that same year, as an estimation, it would take around 13 and 47 years to meet the demand for complete dentures and other prosthetics, respectively, in the Brazilian population aged 65–74 years^15^. Twelve years after the last National Oral Health Survey, 2,140,745 complete dentures, and 619,118 other prosthetics were produced, and since 2010, the Brazilian older population has increased by 55.47% (2021)^5^. Despite the growing production of prostheses over the years being an optimistic finding, the values found are still far below the needs of the Brazilian population.

On March 11, 2020, the World Health Organization declared the situation of the covid-19 pandemic caused by the new coronavirus (SARS-CoV-2)^28^. Worldwide, governments took preventive measures, considered effective the social distancing adopted by the population, and recommended the implementation of social distancing measures^29^, directly impacting health care, especially in the public service. Several countries have decreed the suspension of services considered non-essential, including elective or routine dental care^30^. In March 2020, the Brazilian MS suspended several benefits in the SUS, including elective dental care and maintaining dental emergency care^16^. For the services of CEOs and LRPDs, the MS highlighted in June the role of the local manager in deciding on the operation of services based on local epidemiological characteristics^31^. In this scenario, between 2019 and 2020, there was a reduction of 42.5% in emergency services in primary care and 44.1% in specialized care^32^. Overall, non-urgent procedures decreased by 92.3% from 2019 to 2020, and non-emergency specialist dental care visits decreased by 68.2%^32^. These data confirm the percentage differences in the estimated and performed production of prosthetics for 2020. Given the continuity of the pandemic, maintaining the balance between supply and reducing damage from the long postponement of dental care has become essential. In November 2020, several incentive measures, such as guidance guides and financial incentives, were carried out to assist managers in resuming procedures^33^. Vaccination against the coronavirus began in Brazil in January 2021. These events would reinforce the resumption of production in that country, although below the expected values if no pandemic existed.

Given the results obtained, in conclusion, the policies aimed at the production of prosthetics have been gaining strength and growth over the years. However, production remains very far from the needs of the Brazilian older population, highlighting the lack of equity in providing services between prosthetic types and regions of the country. The covid-19 pandemic negatively impacted the production of dental prosthetics by SUS, and the reduction in output in 2020 was expected, given the prolonged suspension of elective care. Incentive measures and the introduction of vaccination against covid-19 favored the resumption of dental care in 2021, demonstrating a partial recovery in the prosthesis production rate. The federal government must design incentives to recover this deficit and expand access due to the pent-up demand due to the pandemic. The 2020 National Oral Health Survey, expected to be completed in 2023, will be of great importance for the continuity of this study, as it will provide updated data on the need for prosthetics in the country. Analyses of trends in the production of prosthetics in a less comprehensive way, focusing on the Brazilian states, can point out more precisely the strengths and weaknesses, helping and guiding state managers in planning actions in collective oral health. Expanding prosthetic rehabilitation services requires coordination, evaluation, and financing of public policies, with the integrality in the approached strategies being an essential feature. The joint work of prevention actions will prevent the high percentage of edentulousness among older people from persisting with the future universalization of prosthetic and rehabilitative services.
