# Supplementary material for: Production of dental prosthetics in the SUS in Brazilian older population and impact of the covid-19 pandemic
Source: Rev Saude Publica. 2023 Aug 3;57:51. doi: 10.11606/s1518-8787.2023057004828 (PMC10421609; doi:10.11606/s1518-8787.2023057004828)
Supplement: Translation

# Translation: Produção de próteses dentárias no SUS em idosos brasileiros e impacto da pandemia covid-19

**Authors:** Manuella Fluck Vieira, Patrícia Schunck Alferes Marques, Daniela de Rossi Figueiredo, Daniela Lemos Carcereri, Andreia Morales Cascaes

**Affiliations:** I Brasil Universidade Federal de Santa Catarina. Programa de Pós-graduação em Odontologia. Florianópolis, SC, Brasil; II Brasil Faculdade São Leopoldo Mandic. Programa de Pós-graduação em Odontologia. Campinas, SP, Brasil; III Brasil Universidade do Sul de Santa Catarina. Departamento de Odontologia. Florianópolis, SC, Brasil; IV Brasil Universidade Federal de Santa Catarina. Departamento de Odontologia. Florianópolis, SC, Brasil; V Brasil Universidade Federal de Santa Catarina. Departamento de Saúde Pública. Florianópolis, SC, Brasil

**Keywords:** Prótese Dentária, Sistema Único de Saúde, Idoso, Brasil, COVID-19

## Abstract

**OBJETIVO:**

Descrever as tendências na produção de próteses odontológicas pelo Sistema Único de Saúde (SUS) em idosos de 60 anos ou mais no Brasil e nas regiões do país de 2010 a 2019, e o impacto da pandemia de covid-19 na produção esperada para 2020 e 2021.

**MÉTODOS:**

Estudo de séries temporais que utilizou dados secundários provenientes do banco de dados do SUS (Datasus–Tabnet), e do Instituto Brasileiro de Geografia e Estatística, no período de 2010 a 2021. Calcularam-se as taxas padronizadas por idade para o Brasil e regiões de cada ano analisado. Regressões lineares generalizadas, pelo método de estimação de Prais-Winstein, estimaram as tendências das produções.

**RESULTADOS:**

Houve tendência de crescimento na taxa padronizada de produção das próteses totais e das demais próteses para cada 100 mil habitantes, no Brasil e em todas as regiões do país. O aumento na produção de próteses totais foi maior na região Nordeste (50,3%/ano) e menor na região Norte (19,1%/ano). As tendências na produção das demais próteses foram maiores na região Sudeste (120,7%/ano) e menores na região Norte (24,5%/ano). A produção de próteses para ambos os grupos diminuiu nos anos de pandemia. Em 2020, a diferença relativa variou de -36,4% (Norte) até -61,7% (Nordeste) para a produção de próteses totais e de -17,9% (Norte) até -68,4% (Nordeste) para as demais próteses. Em 2021, as taxas padronizadas e a produção absoluta aumentaram quando comparadas ao ano anterior, todavia, em relação aos valores esperados, as diferenças foram próximas às encontradas em 2020.

**CONCLUSÃO:**

Políticas voltadas para a produção de próteses totais e das demais próteses vêm tomando forças e crescendo. Todavia, a produção se mantém distante da necessidade da população e não há equidade na oferta dos serviços. A pandemia de covid-19 impactou negativamente na produção de próteses dentárias pelo SUS.

## INTRODUÇÃO

A perda total dos dentes (edentulismo) ainda é aceita por muitos como um fenômeno natural do envelhecimento^1^. No entanto, representa o desfecho do histórico de tratamentos odontológicos (ou sua ausência) e das doenças dentárias da vida do indivíduo^2^. Apresenta etiologia complexa, com inter-relação de fatores biológicos, sociais e comportamentais, sendo seus principais precursores a cárie e a doença periodontal^3^. Em 2010, 158 milhões de pessoas eram edentadas, constituindo 2,3% da população mundial. Com a idade há aumento gradual da prevalência de perda dentária, com pico de incidência aos 65 anos^4^. O envelhecimento populacional em ritmo acelerado altera a composição etária, impactando os sistemas de saúde.

Os idosos representavam 10,7% da população brasileira em 2010, subindo para 14,3% em 2020^5^. Segundo as Pesquisas Nacionais de Saúde Bucal (SB-Brasil)^6,7^, o percentual da população idosa entre 65 e 74 anos de idade com dentes perdidos manteve-se na faixa de 90% entre os anos de 2003 e 2010. Foi observado na pesquisa de 2010 que, nessa mesma faixa etária, 38,3% necessitavam de prótese total^7^. Ademais, em uma comparação mundial, o Brasil apresentou em 2010 a maior prevalência padronizada por idade de perda dentária severa, com 3,9%^4^. Com relação à qualidade de vida, a perda dentária compromete a rotina diária, traz consequências de natureza funcional e social^8,9^, afeta a autoestima^8^, aumenta o risco de déficit nutricional^9^ e pode refletir nas percepções do indivíduo, incluindo autopercepção da necessidade de tratamento odontológico^10^, além de ser um fator de risco para o envelhecimento cognitivo acelerado^11^. Indivíduos edêntulos sem próteses dentárias apresentam maior impacto negativo na qualidade de vida que os usuários de próteses dentárias^9^.

No Brasil, os serviços de reabilitação protética ocorrem no Sistema Único de Saúde (SUS) por meio das unidades básicas de saúde (UBS)^12^, responsáveis pela atenção primária à saúde, dos centros de especialidades odontológicas (CEOs) e dos laboratórios regionais de próteses dentárias (LRPDs). Cabe aos gestores de saúde analisar a necessidade e a viabilidade e definir a implementação, tipos e quantidade de próteses dentárias a serem produzidas^13^. Existe distribuição desigual na oferta desses serviços no país. Essa desigualdade é confirmada pelas taxas mensais de entrega de próteses, taxas de utilização e oferta dos CEOs e LRPDs nas regiões^14,15^. Em março de 2020, por conta da pandemia de covid-19, vários serviços no SUS, incluindo atendimentos odontológicos eletivos, foram suspensos, mantendo-se apenas o atendimento das urgências odontológicas^16^. Apesar da disponibilidade de dados, faltam estudos sobre a produção protética do SUS em longos períodos e o impacto causado pela pandemia de covid-19 na área. Este estudo objetivou descrever as tendências na produção de próteses odontológicas pelo SUS em idosos de 60 anos ou mais no Brasil e regiões de 2010 a 2019, além de estimar o impacto originado pela pandemia de covid-19 na produção esperada para os anos de 2020 e 2021.

## MÉTODOS

Trata-se de um estudo de séries temporais que utilizou dados secundários provenientes do banco de dados do SUS (Datasus–Tabnet)^17^ e do Instituto Brasileiro de Geografia e Estatística (IBGE)^5^, no período de 2010 a 2021.

A produção de próteses dentárias foi obtida a partir dos registros da Produção Ambulatorial do SUS (SIA/SUS), no site do Datasus–Tabnet^17^, sendo classificada segundo local de residência, faixa etária e ano de processamento dos dados no sistema. Na obtenção dos dados, optou-se por local de residência ao invés de local de atendimento, pois apenas no primeiro é possível a escolha da faixa etária dos usuários. A produção de próteses dentárias registrada considera a quantidade aprovada para pagamento das secretarias, segundo cadastrado no Sistema de Gerenciamento da Tabela de Procedimentos, Medicamentos, Órteses, Próteses e Materiais Especiais do SUS (Sigtap)^18^. Os procedimentos selecionados foram divididos em dois grandes grupos, denominados: grupo das próteses totais (07.01.07.012-9: prótese total mandibular; 07.01.07.013-7: prótese total maxilar) e grupo das demais próteses (07.01.07.009-9: prótese parcial mandibular removível; 07.01.07.010-2: prótese parcial maxilar removível; 07.01.07.014-5: próteses coronárias/intrarradiculares fixas/adesivas por elemento; 07.01.07.015-3: prótese dentária sobre implante).

Os dados coletados referentes à quantidade de idosos residentes nos estados brasileiros foram obtidos na Projeção da População do Brasil e Unidades da Federação por Sexo e Idade para o Período 2000–2030, do IBGE^5^, cujos dados estão disponíveis no site do Datasus–Tabnet^17^. Este estudo considerou idosos os indivíduos com idade igual ou superior a 60 anos^19^.

Para a análise dos dados, a partir da população e produção encontradas no período, foram calculadas as taxas bruta e padronizada por idade, para o Brasil e para cada região nos anos analisados. O cálculo da taxa bruta considerou a produção de próteses dividida pela população real de idosos de cada local multiplicada por 100 mil habitantes. A taxa padronizada por idade usou o método direto^20^, com intervalos de faixa etária de 5 em 5 anos, tomando como padrão a média da estrutura etária da população idosa do país no período analisado multiplicada por 100 mil habitantes.

Os dados foram organizados e analisados em planilhas no programa Microsoft Excel. As ferramentas e fórmulas do próprio programa permitiram gerar tabelas e figuras. A análise de séries temporais da produção das próteses foi conduzida no programa Stata 14.2 (StataCorp LP, College Station, Estados Unidos), por meio de regressões lineares generalizadas, pelo método de estimação de Prais-Winstein^21^. As estimativas das tendências de produção de próteses consideraram o período de 2010 a 2019, tendo em vista que os anos 2020 e 2021 apresentaram queda significativa na produção de próteses, em função da pandemia de covid-19. As seguintes fórmulas foram calculadas para obtenção dos valores de variação percentual anual (VPA) = 100*(-1 + 10^b) e de IC95% = 100*(-1 + 10^[b ± t*EP])^21^. A tendência foi considerada crescente quando os coeficientes foram positivos, decrescente quando negativos e estável quando os coeficientes da regressão não foram significativamente diferentes de zero (p > 0,05). O cálculo da VPA no período de 2010 a 2019 foi utilizado para estimar a produção esperada para 2020 e 2021 se não tivesse havido pandemia. A diferença absoluta entre a taxa de produção observada e esperada para o ano de 2020 considerou a taxa de produção observada em 2020 menos a taxa de produção esperada em 2020. A diferença relativa entre a taxa de produção observada e esperada para o ano de 2020 foi calculada pela fórmula = [(taxa de produção observada em 2020 - taxa de produção esperada em 2020) -1] × 100. Cálculo análogo foi realizado para as estimativas do ano 2021.

## RESULTADOS

A Tabela 1 apresenta a evolução da produção de próteses totais para idosos de 60 anos ou mais, no período de 2010 a 2021, no Brasil e regiões. Houve tendência de crescimento na taxa padronizada de produção das próteses totais para cada 100 mil habitantes, no Brasil e em todas as regiões do país. Esse aumento foi maior na região Nordeste (VPA = 50,3%) e menor na região Norte (VPA = 19,1%). A produção das próteses totais reduziu no ano 2020 e voltou a subir em 2021. A diferença absoluta entre as taxas de produção padronizadas observadas e esperadas para 2020 variou de -333,0 (Norte) até -1.314,1 (Nordeste). A diferença relativa de queda variou de -36,4% (Norte) até -61,7% (Nordeste). Apesar do aumento da taxa padronizada encontrado em 2021, as diferenças absoluta e relativa entre o esperado e o observado nesse ano foram próximas e, em sua maioria, maiores que as encontradas em 2020.

**Tabela 1:** Evolução da produção de próteses totais em idosos de 60 anos ou mais, segundo região do Brasil e no geral. Datasus, Brasil, 2010–2021.

Variáveis	Norte	Nordeste	Sudeste	Sul	Centro-Oeste	Brasil
Taxa padronizada de produção por ano						
2010	331,0	251,7	268,3	424,3	408,1	301,5
2011	380,2	476,6	469,5	539,0	565,4	483,9
2012	460,7	618,0	619,3	624,1	782,1	621,2
2013	546,1	832,5	683,0	623,0	745,9	706,0
2014	582,6	1.188,8	751,5	763,1	918,5	860,9
2015	561,6	1.071,8	777,8	851,9	897,0	856,7
2016	430,0	1.096,7	775,9	878,2	929,4	860,7
2017	439,2	1.049,4	678,3	854,0	809,2	791,1
2018	576,7	1.292,6	667,1	881,7	979,3	865,6
2019	767,3	1.416,8	747,3	943,1	1.078,7	959,2
2020	580,9	815,3	392,5	558,5	715,9	550,2
2021	606,3	985,2	528,7	657,0	840,3	680,8
Regressão de Prais-Winsten (2010 a 2019)						
Coeficiente β1 (IC95%)	0,076 (0,009– 0,142)	0,177 (0,077– 0,277)	0,101 (0,005– 0,198)	0,084 (0,050– 0,118)	0,089 (0,037– 0,141)	0,119 (0,039– 0,199)
VPA (IC95%)	19,1 (2,2– 38,8)	50,3 (19,5– 89,1)	26,3 (1,1– 57,7)	21,4 (12,2– 31,3)	22,7 (8,8– 38,5)	31,5 (9,5– 58,0)
Valor-p	0,030	0,003	0,042	0,000	0,004	0,009
Produção esperada para 2020						
Taxa de produção esperada	913,9	2.129,5	943,8	1.144,8	1.323,9	1.261,7
Diferença absoluta (taxa)	-333,0	-1.314,1	-551,3	-586,2	-608,0	-711,5
Diferença relativa (%)	-36,4	-61,7	-58,4	-51,2	-45,9	-56,4
Produção esperada para 2021						
Taxa de produção esperada	1.088,5	3.200,6	1.192,0	1.389,6	1.625,0	1.659,7
Diferença absoluta (taxa)	-482,2	-2.215,4	-663,3	-732,6	-784,7	-978,9
Diferença relativa (%)	-44,3	-69,7	-55,6	-52,7	-48,3	-59,0

IC95%: intervalo de confiança de 95%; VPA: variação percentual anual.

Nota: taxa de produção padronizada pelo método direto. Taxa de produção esperada para 2020 = [(taxa de produção de 2019 × VPA)/100] + taxa de produção de 2019. Diferença absoluta = taxa de produção observada em 2020 - taxa de produção esperada em 2020. Diferença relativa = [(taxa de produção observada em 2020 - taxa de produção esperada em 2020) - 1] × 100. Cálculo análogo foi realizado para as estimativas do ano 2021.

A Tabela 2 trata da evolução da produção das demais próteses em idosos de 60 anos ou mais, no período de 2010 a 2021, no Brasil e regiões. A taxas padronizadas de produção aumentaram com o decorrer dos anos, exceto para a região Norte, a qual apresentou estabilidade. A região Sudeste (VPA = 120,7%) e a Nordeste (VPA = 92,1%) apresentaram as maiores tendências de aumento na produção das demais próteses. A diferença absoluta entre as taxas padronizadas observadas e esperadas para os anos de 2020 foi maior nas regiões Nordeste (-550,5) e Centro-Oeste (-620,6). A diferença relativa variou de -17,9% (Norte) até -68,4% (Nordeste). Para o ano de 2021 as diferenças aumentaram. Os maiores valores absolutos encontrados foram: Nordeste (-1.208,2) e Centro-Oeste (-1.656,8). A diferença relativa variou de -33,2% (Norte) até -82,4% (Centro-Oeste).

**Tabela 2:** Evolução da produção das demais próteses em idosos de 60 anos ou mais, segundo região do Brasil e no geral. Datasus, Brasil, 2010–2021.

Variáveis	Norte	Nordeste	Sudeste	Sul	Centro-Oeste	Brasil
Taxa padronizada de produção por ano						
2010	67,7	31,3	53,6	49,7	13,9	45,5
2011	97,5	59,4	81,6	105,5	53,2	79
2012	120,3	82,8	113,8	182	142,9	119,5
2013	158,2	145,5	137,1	213	187,1	155,8
2014	108,4	240,9	167,4	269	213,1	201,5
2015	94,8	245,1	197,9	258,5	263,8	217,8
2016	68,5	278,5	218	309,5	294	244,2
2017	95	300,8	210,6	326,7	259,4	247,9
2018	139	368,1	227,8	354,3	362,8	285,5
2019	215	418,8	263,2	398,8	412,7	328,9
2020	219,9	254	147,6	253,7	290,2	203,5
2021	222,7	337,2	216,6	297,1	353	267,5
Regressão de Prais-Winsten (2010 a 2019)						
Coeficiente β1 (IC95%)	0,095 (-0,032 a 0,223)	0,284 (0,166 a 0,401)	0,174 (0,099 a 0,248)	0,214 (0,100 a 0,329)	0,344 (0,130 a 0,557)	0,215 (0,119 a 0,311)
VPA (IC95%)	24,5 (-7,2 a 67,1)	92,1 (46,5 a 151,9)	49,1 (25,5 a 77,1)	63,8 (25,9 a 113,2)	120,7 (35,0 a 260,7)	64,1 (31,7 a 104,5)
Valor-p	0,124	0,001	0,001	0,003	0,006	0,001
Produção esperada para 2020						
Taxa de produção esperada	267,7	804,5	392,5	653,3	910,8	539,7
Diferença absoluta (taxa)	-47,8	-550,5	-244,9	-399,6	-620,6	-336,2
Diferença relativa (%)	-17,9	-68,4	-62,4	-61,2	-68,1	-62,3
Produção esperada para 2021						
Taxa de produção esperada	333,3	1.545,4	585,3	1.070,2	2.009,8	885,6
Diferença absoluta (taxa)	-110,6	-1.208,2	-368,7	-773,1	-1.656,8	-618,1
Diferença relativa (%)	-33,2	-78,2	-63	-72,2	-82,4	-69,8

IC95%: intervalo de confiança de 95%; VPA: variação percentual anual.

Nota: taxa de produção padronizada pelo método direto. Taxa de produção esperada para 2020 = [(taxa de produção de 2019 × VPA)/100] + taxa de produção de 2019. Diferença absoluta = taxa de produção observada em 2020 - taxa de produção esperada em 2020. Diferença relativa = [(taxa de produção observada em 2020 - taxa de produção esperada em 2020) - 1] × 100. Cálculo análogo foi realizado para as estimativas do ano 2021.

As Figuras 1 e 2 apresentam as produções absolutas de próteses totais e demais próteses, respectivamente, no período de 2010 a 2021, no Brasil e suas regiões. A produção nacional de próteses totais apresentou crescimento quase constante, iniciando com 59.134 próteses produzidas em 2010, atingindo 269.886 em 2019, caindo para 161.144 em 2020 e voltando a subir em 2021, com 207.547 próteses totais (Figura 1). A produção nacional das demais próteses apresentou crescimento até 2019, iniciando com 8.916 próteses produzidas em 2010, atingindo 92.499 em 2019, caindo para 59.504 em 2020 e retornando o aumento em 2021, com 81.311 próteses (Figura 2). Na maior parte dos anos, a produção absoluta de ambos os grupos analisados foi menor na região Norte e maior na Sudeste.

**Figura 1:** Produção absoluta de próteses totais em idosos de 60 anos ou mais, segundo região do Brasil e no geral. Datasus, Brasil, 2010–2021.

**Figura 2:** Produção absoluta das demais próteses em idosos de 60 anos ou mais, segundo região do Brasil e no geral. Datasus, Brasil, 2010–2021.

## DISCUSSÃO

Este estudo buscou analisar as tendências de produção de próteses dentárias pelo SUS em idosos de 60 anos ou mais no Brasil e regiões e, ainda, observar o impacto causado pela pandemia de covid-19. Os principais resultados apontam crescente produção de próteses no país até 2019, ano anterior à pandemia. O grupo de próteses totais apresentou maior produção quando comparado ao grupo das demais próteses. No Brasil e em todas as regiões, em ambos os grupos analisados, foi significativo o impacto da pandemia na redução da produção protética.

Neste estudo utilizaram-se e valorizaram-se dados públicos e disponíveis, a partir dos quais foram produzidos conhecimentos relevantes para a gestão de saúde bucal pública de abrangência nacional. Quanto às limitações do estudo, destaca-se o banco de dados utilizado, pois o SIA-SUS não possui padronização dos registros e ainda faltam estudos que avaliem a confiabilidade dos dados referentes aos procedimentos odontológicos. O sistema muitas vezes é utilizado para faturamento dos procedimentos realizados, levando à possibilidade de sobrerregistro.

Em relação ao aumento anual da produção absoluta de próteses, os resultados estão relacionados às políticas públicas voltadas para a expansão da oferta desses procedimentos. Inserida em 2000 no Programa Saúde da Família (PSF), a odontologia prestava assistência de baixa resolutividade, dependendo de serviços meramente curativos e mutiladores^22^. Em 2004, com a implementação do programa Brasil Sorridente, houve organização e planejamento dos serviços de saúde bucal^22^. Até o lançamento do programa, apenas 2,8% dos procedimentos odontológicos realizados no SUS eram tratamentos especializados^23^. Desde então, os atendimentos de instalação e manutenção das próteses podem ser realizados na atenção primária à saúde^12^ e nos CEOs e a produção das próteses nos LRPDs e CEOs, com exceção das próteses sobre implante, que ocorrem apenas no último^24^. Em 2009, a portaria de critérios, normas e requisitos para a implantação e habilitação dos CEOs e LRPDs tinha cinco anos de criação^13^. É importante observar que, no geral, além do crescimento anual da produção absoluta, as taxas padronizadas também aumentaram com o passar do tempo.

Os achados deste estudo apontam para as desigualdades na produção de próteses entre as regiões brasileiras. Diversos fatores podem estar relacionados a isso, como: o modo de implementação e gestão dos LRPDs e CEOs^13,25^, a quantidade de unidades credenciadas^26^ e o número mínimo de procedimentos realizados^13^, assim como as diferenças socioeconômicas, demográficas^2^ e de necessidade das regiões^7^. A região Norte, seguida da Centro-Oeste, apresenta as menores quantidades de CEOs e LRPDs^15,26^. O Nordeste apresenta os maiores números, seguido do Sudeste^15,26^. Esses dados corroboram parcialmente a produção absoluta encontrada neste estudo, principalmente no que se refere à produção de próteses totais.

A data de inserção dos tipos de procedimento e seus valores ambulatoriais podem interferir nas diferenças encontradas entre os grupos de próteses analisados. Por exemplo, a prótese sobre implante passou a ser ofertada apenas no fim de 2010^18^ e, ainda, esse tipo de tratamento pode ser realizado somente nos CEOs, cabendo ao gestor municipal solicitar via ofício ao Ministério da Saúde (MS) para oferecer esses serviços^27^. Além da maior complexidade envolvida na produção das demais próteses, questões referentes ao custo dos procedimentos podem explicar a menor produção desse grupo. A prótese sobre implante, desde sua implementação no sistema, tem valor de R$ 300,00, e os demais procedimentos, desde 2012, têm o valor de R$ 150,00^18^. Todavia, sabe-se que o custo laboratorial é diferente entre as próteses e a prótese total geralmente apresenta menor custo, facilidade de instalação, produção e manutenção, além de maior grau de severidade da condição bucal, fatores que podem estar relacionados à maior produção encontrada nesse grupo. Sobre a produção mínima de procedimentos realizados, a maioria dos LRPDs do país estão credenciados com produção entre 20 e 50 próteses por mês^26^. A especialidade de prótese dentária não está prevista como obrigatória nos CEOs^13^. Esses fatores corroboram as baixas taxas padronizadas de produção encontradas neste estudo.

Segundo dados do SB Brasil 2010^7^, as necessidades de prótese variam entre as regiões e grupos analisados. Para a necessidade de prótese total, do maior para o menor, os seguintes percentuais foram identificados no último levantamento nacional: 46,5% (Centro-Oeste), 45,6% (Norte), 41,1% (Nordeste), 39% (Sudeste) e 27,3% (Sul). Essas necessidades, quando relacionadas aos resultados deste estudo, demostram desigualdade da produção, principalmente em relação à região Norte. Com relação às demais próteses, os percentuais de necessidade foram: 66,1% (Sul), 61,7% (Nordeste), 58% (Sudeste), 56,4% (Centro-Oeste) e 56,3% (Norte)^7^. Apesar de apresentar maiores necessidades, o grupo das demais próteses tem menor produção. Ainda, a quantidade de LRPD por região não segue a ordem de necessidade de prótese dentária^14^. Vale ressaltar que as diferenças regionais, em ambos os grupos, tendem a aumentar, direcionando-se para maior contraste, devido ao aumento da produção não seguir a ordem de necessidade.

Em parâmetros nacionais, 38,3% dos idosos de 65–74 anos de idade necessitavam de prótese total e 59,3% de algum dos tipos do grupo das demais próteses^7^, percentuais que representavam em números absolutos no ano de 2010, conforme a Projeção da População do Brasil e Unidades da Federação por Sexo e Idade para o Período 2000–2030, respectivamente, 7.507.510 e 11.623.899 idosos brasileiros com 60 anos ou mais. Nesse mesmo ano, estimou-se ser necessário em torno de 13 e 47 anos para suprir a demanda próteses totais e demais próteses, respectivamente, na população brasileira de 65–74 anos^15^. Decorridos 12 anos do último levantamento nacional de saúde bucal, foram produzidas 2.140.745 próteses totais e 619.118 das demais próteses e, desde 2010, a população idosa brasileira aumentou 55,47% (2021)^5^. Observa-se que, apesar da crescente produção de próteses no decorrer dos anos ser um achado otimista, os valores encontrados ainda estão muito aquém das necessidades da população brasileira.

Oficializou-se pela Organização Mundial da Saúde dia 11 de março de 2020 a situação de pandemia de covid-19, causada pelo novo coronavírus (SARS-CoV-2)^28^. Medidas preventivas foram tomadas, o distanciamento social adotado pela população foi considerado efetivo e a implementação de medidas de distanciamento social foi recomendada^29^, impactando diretamente os atendimentos de saúde, principalmente no serviço público. Diversos países decretaram a suspensão de serviços considerados não essenciais, incluindo os atendimentos odontológicos eletivos ou de rotina^30^. Em março de 2020, o MS brasileiro suspendeu vários serviços no SUS, incluindo atendimentos odontológicos eletivos, mantendo-se o atendimento das urgências odontológicas^16^. Para os serviços dos CEOs e LRPDs, o MS ressaltou em junho o papel do gestor local para decisão sobre o funcionamento dos serviços, baseado nas características epidemiológicas locais^31^. Nesse panorama, entre 2019 e 2020, observou-se redução de 42,5% em serviços de urgência na atenção primária e de 44,1% na atenção especializada^32^. No geral, os procedimentos não urgentes diminuíram 92,3% de 2019 a 2020, e consultas de atendimento odontológico especializado não emergencial diminuíram 68,2%^32^. Esses dados confirmam as diferenças percentuais na produção estimada e realizada de próteses para 2020. Diante da continuidade da pandemia, a manutenção do equilíbrio entre oferta e redução dos prejuízos do adiamento prolongado da assistência odontológica tornou-se imprescindível. Em novembro de 2020, diversas medidas de incentivo, como guias de orientação e portarias de incentivos financeiros, foram realizadas a fim de auxiliar os gestores na retomada dos procedimentos^33^. A vacinação contra o coronavírus iniciou-se no Brasil em janeiro de 2021. Esses acontecimentos reforçam a retomada da produção nesse, ainda que abaixo dos valores esperados caso não houvesse pandemia.

Diante dos resultados obtidos conclui-se que no decorrer dos anos as políticas voltadas para a produção de próteses vêm tomando força e crescendo. Todavia, a produção se mantém muito distante da necessidade da população idosa brasileira, destacando-se a falta de equidade na oferta dos serviços entre os tipos protéticos e regiões do país. A pandemia de covid-19 impactou negativamente a produção de próteses dentárias pelo SUS e a redução na produção de 2020 era esperada, dada a prolongada suspensão dos atendimentos eletivos. As medidas de incentivo e a introdução da vacinação contra covid-19 favoreceram a retomada dos atendimentos odontológicos, em 2021, demonstrando recuperação parcial na taxa de produção das próteses. É necessário que o governo federal projete incentivos para recuperar este déficit e ampliar o acesso em função da demanda reprimida pela pandemia. A Pesquisa Nacional de Saúde Bucal de 2020, prevista para ser finalizada em 2023, terá grande importância para continuidade deste estudo, pois trará dados atualizados da necessidade de prótese no país. Estudos das tendências na produção de próteses de maneira menos abrangente, com foco nos estados brasileiros, podem apontar de maneira mais precisa os pontos fortes e fracos, auxiliando e orientando os gestores estaduais no planejamento de ações no âmbito da saúde bucal coletiva. É preciso haver coordenação, avaliação e financiamento das políticas públicas para a ampliação da oferta de serviços de reabilitação protética, sendo imprescindível a integralidade nas estratégias abordadas. Com o trabalho conjunto de ações de prevenção, poder-se-á evitar que o elevado percentual de edentulismo entre idosos perdure e que no futuro haja universalização dos serviços protéticos reabilitadores.
